# Self-rated impulsivity in healthy individuals, substance use disorder and ADHD: psychometric properties of the Swedish Barratt impulsiveness scale

**DOI:** 10.1186/s12888-021-03462-1

**Published:** 2021-09-18

**Authors:** Lotfi Khemiri, Christoffer Brynte, Maija Konstenius, Joar Guterstam, Ingvar Rosendahl, Johan Franck, Nitya Jayaram-Lindström

**Affiliations:** Department of Clinical Neuroscience, Centre for Psychiatry Research, Karolinska Institutet & Stockholm Health Care Services, Norra Stationsgatan 69, floor 7, 113 64 Stockholm, Sweden

**Keywords:** BIS, Barratt impulsiveness scale, Impulsivity, Motor impulsivity, Non-planning impulsivity, Attentional impulsivity, Psychometrics, Substance use disorder, Alcohol, Amphetamine, ADHD

## Abstract

**Background:**

Impulsivity is associated with several psychiatric disorders, including substance use disorders (SUD) and attention deficit hyperactivity disorder (ADHD). A widely used questionnaire to assess impulsivity is the Barratt Impulsiveness Scale (BIS), and the aim of the current study was to evaluate the psychometric properties of the Swedish version of the BIS (swe-BIS).

**Methods:**

The original BIS was translated to Swedish and back-translated by an authorized translator. The swe-BIS was administered to healthy controls (*n* = 113), patients with alcohol use disorder (*n* = 97), amphetamine use disorder (*n* = 37) and attention deficit hyperactive disorder (ADHD; *n* = 26). A subset of subjects (*n* = 62) completed the swe-BIS twice within 1 week. Psychometric evaluation of the swe-BIS included assessment of different indices of reliability (internal consistency, test-retest and agreement) and validity (response processess, divergent and convergent). Confirmatory factor analyses (CFA) were performed to assess several indices of model fit in five different models based on previously suggested subscales.

**Results:**

Cronbach’s alpha for all swe-BIS items in the full sample was 0.89, ranging from 0.78–0.87 within the different subgroups. The Pearson test-retest correlation for total score was 0.78 (*p* < 0.001), with greater test-retest correlations within compared to across different subscales. The Bland-Altman plot indicated high level of agreement between test and retest. The healthy individuals had lower swe-BIS score compared to the patients (t(267.3) = − 8.6; *p* < 0.001), and the swe-BIS total score was also significantly different between each of the four participant groups (*p* < 0.01 for all group comparisons). Furthermore, swe-BIS had greater correlations with impulsivity related scales compared to non-impulsivity related scales. The CFA analyses indicated that while no suggested model showed an optimal fit, the best model fit indices was found for the 3-factor model.

**Conclusions:**

The swe-BIS was found to have good to excellent psychometric properties with respect to the assessed indices of reliability and validity, supporting use of the scale in clinical research in both healthy individuals and patients with SUD and ADHD.

**Supplementary Information:**

The online version contains supplementary material available at 10.1186/s12888-021-03462-1.

## Introduction

Impulsivity is a heterogenous personality and behavioural construct [1], present in the general population as well as in several psychiatric disorders, including substance use disorders (SUD), personality disorders and attention deficit hyperactivity disorder (ADHD). An impulsive act is in essence characterized by haste, lack of premeditation and disregard of the future consequences of the action. Even though no formal definition exists, a widely held definition presented by Moeller and colleagues is that impulsivity is “a predisposition toward rapid unplanned reactions to internal or external stimuli without regard to the negative consequences of these reactions to the impulsive individual or to others” [2].

There are a wide number of different scales and tests used to measure the construct of impulsivity. A distinction is often made between trait and state impulsivity: State impulsivity varies across time, is influenced by short-term experimental conditions and can be assessed by different laboratory based neuropsychological tests. Such tests aim to capture an individual’s propensity toward impulsive behaviours, e.g., inability to inhibit prepotent responses, ability to plan for the future and the valuing of delayed rewards [3]. Trait impulsivity, or impulsiveness, in contrast, refers to the overall degree of impulsive behaviour in an individual that is relatively constant across time. This trait is instead assessed by self-rating questionnaires, where the subject is asked to rate how impulsive they are in general across several different behaviours and life situations. Common instruments used to assess trait impulsivity are the Impulsivity-Venturesomeness-Empathy scale [4], the Urgency, Premeditation, Perseverance, Sensation Seeking, Positive Urgency, Impulsive Behavior Scale [5] and the Barratt Impulsiveness Scale (BIS [6];).

The BIS is one of the most widely used self-rating questionnaires for impulsiveness. As reviewed by Stanford and colleagues, the BIS was developed by Barratt who originally conceptualized impulsiveness as a multidimensional construct which was orthogonal to anxiety [7]. After a series of revisions this work resulted in the BIS-11, which is the most recent version of the scale where impulsiveness is treated as a multidimensional construct [6]. Based on principal components analysis of the BIS-11, the trait of impulsiveness was conceptualized by Patton, Stanford and Barratt as consisting of six first-order factors (attention, cognitive instability, motor, perseverance, self-control, cognitive complexity) which in turn formed three second-order factors entitled motor, attentional and non-planning impulsiveness. However, this model has been questioned given that other psychometric studies of the BIS has found better fit for two-factor solutions [8], while other studies have found best fit for three factor solutions, but with different item loadings compared to the original study [9]. In a systematic review of the psychometric properties of the BIS-11, it was concluded that even though the BIS-11 is a useful clinical tool to differentiate different clinical populations with high levels of impulsivity, there are conflicting data regarding the dimensions/subscales of the scale [10].

The BIS has been extensively used in clinical research and practice during the last decades [7], including studies of clinical populations with affective disorders [11, 12], prison inmates [13], antisocial personality disorder [14] and ADHD [15]. Furthermore, BIS scores have also been found to be elevated in patients with different forms of SUD, including alcohol [16, 17], cocaine [18] and opioids [19]. The BIS has been translated to other languages, including German [20, 21], Spanish [22], Norwegian [23], Chinese [24], Italian [9], Portuguese [25], Dialectal Arabic [26] and Japanese [27]. Considering the wide interest for impulsivity in clinical psychiatric research, we decided to translate the scale into Swedish. Because of the conflicting results of earlier studies, we also wanted to explore its psychometric properties in different populations.

The aim of the current was thus to perform a psychometric evaluation of a Swedish version of the BIS-11 (swe-BIS), including analyses of reliability, validity and confirmatory factor analyses in patients with SUD, ADHD and healthy volunteers. The main hypothesis was that the patient populations would exhibit elevated levels of impulsiveness compared to the control group (evidence of validity based on response processes). In addition we hypothesized that the swe-BIS outcomes should correlate to a higher degree with other impulsivity scales, and that the scale would have good test-retest and internal consistency properties.

## Methods

### Adaptation of the BIS

The English version of the BIS-11 [6] was translated and back-translated from English to Swedish by an authorized bilingual translator. A meeting was held with clinicians and clinical researchers to examine the individual items, and minor adjustments regarding exact phrasing of the items were discussed and agreed upon through consensus within the group. See supplementary material Table S1 for the Swedish translation of all BIS items.

### Participants

The swe-BIS was administered to four groups of participants. All individuals were included in different research projects and were administered the swe-BIS as part of the baseline assessment. The first group was patients with alcohol use disorder (AUD) that were administered the questionnaire while taking part in two separate clinical treatment studies [28, 29]. The second group was patients with amphetamine use disorder (AMPH) who completed the questionnaire when participating in a pharmacological fMRI study [30]. The third group comprised subjects with ADHD who were taking part in a pharmacological laboratory experimental study (Brynte et al., in preparation). The final group was healthy controls (HC) with no SUD or any other psychiatric or somatic illnesses, who were recruited from the community, to serve as control group to the AUD patient population in a separate study of cognitive functioning [31]. For each of the aforementioned studies, ethics approval was obtained from the Regional Ethics Review Board in Stockholm, and conducted in accordance with Good Clinical Practice and the declaration of Helsinki.

### Inclusion and exclusion criteria

The major inclusion and exclusion criteria have been described in detail in the original studies [28–31]. In brief, the main inclusion criteria for the patients were current DSM-IV diagnosis of SUD (AUD or AMPH) or ADHD, no severe psychiatric disorder (e.g., bipolar disorder or schizophrenia), no severe somatic illness and no current suicidal ideation. All patients were required to be sober and drug free (assessed by alcometer and urine dip test) on test day, except for the subgroup of AMPH patients who were allowed to have positive urine dip test of central stimulants on the test day. For the HC group, the inclusion and exclusion criteria were similar except that they could not have had any current diagnosis or history of SUD, no illicit drug use the past 12 months, be sober and leave negative urine dip test for drugs of abuse on the day of testing.

### Procedure

All participants underwent a medical evaluation by an M.D., including physical and psychiatric assessment using the Structured Clinical Interview for DSM-IV [32] or the Swedish DSM-5 version of the Mini-International Neuropsychiatric Interview [33]. The ADHD patients already had an existing ADHD diagnosis and confirmed by assessment of patient chart data. Alcohol, nicotine and drug consumption was assessed by the Time Line Follow Back (TLFB) interview [34]. All participants performed a breathalyzer test and urine dip test on the day of testing, and completed several self-rating questionnaires as part of baseline assessment before study participation.

### Questionnaires

#### Barratt impulsiveness scale, Swedish version

The Swedish version of the Barratt Impulsiveness Scale (swe-BIS) comprises all the original 30 items from the original English BIS-11 [6]. Each item is a statement about impulsivity related thoughts/behaviours in different situations, and the subject is asked to rate how often he or she experiences such thoughts or behaviours on a 4-point scale (1 = “Rarely/never”, 2 = “Occasionally”, 3 = “Often” and 4 = “Almost always/always”). The BIS has three subscales namely motor, attention and non-planning impulsiveness. Examples of items from each of the subscales are “I make up my mind quickly” (Motor impulsiveness), “I often have extraneous thoughts when thinking “(Attentional impulsiveness) and “I say things without thinking “(Non-planning impulsiveness).

The swe-BIS was administered to a majority of participants in paper form, and the subjects were asked to complete the questionnaire using a pen. A minority of patients (ADHD group; *n* = 26) completed the same questionnaire through an electronic case record form (CRF) software and used a mouse to enter the responses on the computer screen. A subsample of subjects (*n* = 62) completed the swe-BIS using pen and paper at home approximately 1 week prior to the test session.

#### Montgomery-Åsberg depression self-rating scale

The Montgomery-Åsberg Depression Self-Rating Scale (MADRS-S [35];) is a self-rating instrument consisting of 9 items regarding depression symptoms during the last 3 days. The items are rated on a scale from 0 to 6, so the total score ranges from 0 to 54, with higher scores indicating more severe levels of depressive symptoms.

#### Adult ADHD self-report scale

The World Health Organization Adult ADHD Self-report Scale (ASRS [36];) is a screening questionnaire for adult ADHD according to the DSM-IV criteria. It consists of 18 questions (e.g., “How often do you have problems remembering appointments or obligations?”) which are rated on a scale from 0 (never) to 4 (very often), resulting in a total score and two subscales of hyperactivity and inattention.

#### Obsessive compulsive drinking scale

The Obsessive Compulsive Drinking Scale (OCDS) is a widely used scale assessing compulsions and obsessions related to alcohol craving and drinking [37]. It consists of 14 items (e.g., “How much of your time when you’re not drinking is occupied by ideas, thoughts, impulses, or images related to drinking?”) which are rated from 0 to 4 resulting in a total sum score as well as two subscales of obsessions and drinking control/consequences.

#### Swedish universities scales of personality

The Swedish Universities Scales of Personality (SSP [38];) is a personality test, originally based on the Karolinska Scales of Personality, consisting of 91 individual items. Each item is a statement which is rated on a four-point-scale from ‘does not apply at all’ to ‘applies completely’. The items are subdivided into 13 subscales e.g., impulsiveness and social desirability.

### Statistical analysis

Sociodemographic and clinical background variables are presented as mean ± standard deviation and fractions for continuous and categorical outcomes, respectively. Internal consistency was assessed by calculating Cronbach’s alpha for all BIS items and each subscale, both in the total patient sample and within each of the different patient groups. Test-retest reliability was investigated by calculating Pearsons correlation, for total score and subscale scores, between the two time points of administration. To visually assess agreement between test-retest scores, Bland-Altman plots were created. In these plots, the X axis shows the mean difference between test sessions and the Y axis shows the average score across both test sessions, and it is expected that a majority of the difference scores would fall within + − 2 (i.e., 1.96) standard deviations of the mean difference [39].

Validity is a complex unitary construct for which there are different forms of evidence. Evidence of validity based on response processes (commonly referred to as construct validity) was evaluated by a series of statistical tests where group was the independent variable and mean score of the swe-BIS total score and subscales were the dependent variables. First, t-tests were used to compare healthy controls and all patients on the swe-BIS outcomes. Second, a oneway-ANOVA with group (HC, AUD, AMPH, ADHD) as independent variable was performed. Planned paired comparisons using t-tests without adjustment for multiple comparisons were performed between each group category. Because of lack of homogeneity of variances across groups assessed by Levene’s test, Welch t-test and ANOVA was used. Evidence of convergent validity was evaluated by calculating Pearsons correlation between swe-BIS and ASRS total score, ASRS hyperactive, ASRS inattention and the SSP subscale of impulsivity. Finally, evidence of divergent validity in contrast, was investigated by correlating the swe-BIS to MADRS-S, OCDS and the SSP subscale of social desirability.

In the Confirmatory Factor Analyses (CFA), Mardia’s Multivariate Normality Test was used to test the assumption of multivariate normality in the data. Comparison in model fit to data between several models was done by following fit measures: Chi-square value, root mean squared error of approximation (RMSEA), standardized root mean squared residual (SRMR), comparative fit index (CFI), Tucker-Lewis index (TLI) and Akaike’s information criterion (AIC). Five models of the swe-BIS were tested: a single-factor (total score) model, three versions of a 2-factor model and finally a 3-factor model, based on the three BIS subscales of attentional, motor and non-planning impulsiveness.

All statistical analyses were performed using R [40] and the following packages: psych [41], BlandAltmanLeh [42], MVN [43], lavaan [44], semTools [45] and plyr [46].

## Results

### Study participants

In total 273 participants consisting of HC (*n* = 113), patients with AUD (*n* = 97), AMPH (*n* = 37) and ADHD (*n* = 26), completed the swe-BIS questionnaire and were thus included in the study. Table 1 presents the sociodemographic and clinical background variables in detail for all participants and for each subgroup.

**Table 1:** Sociodemographic and clinical background variables in the full sample, consisting of healthy controls and patients with amphetamine use disorder (AMPH), alcohol use disorder (AUD) and attention deficit hyperactive disorder (ADHD). Values are presented as mean (standard deviation) unless stated otherwise

	Full sample (*n* = 273)	Healthy controls (*n* = 113)	AMPH (*n* = 37)	AUD (*n* = 97)	ADHD (*n* = 26)
Age	45.5 (10.4)	45.8 (11.5)	44.4 (9.9)	47.7 (7.6)	36.9 (11.2)
N (%) females	109 (40.0%)	47 (41.6%)	0 (0%)	45 (46.4%)	17 (65.4%)
School years ^a^	13.3 (3.3)	16.2 (2.5)	10.8 (3.2)	13.8 (2.6)	12.7 (2.1)
Nicotine daily use	40%	11.5%	86.5%	60.0%	26.9%
OCDS total ^b^	15.5 (11.8)	2.1 (2.5)	NA	23.8 (6.4)	NA
% drinking days ^c^	45 (34)	13 (12)	NA	72 (21)	NA
% amphetamine use days	9.9 (28)	0	73 (35)	0	0
MADRS-S ^d^	6.5 (6.6)	3.0 (3.3)	NA	7.6 (6.5)	11.5 (8.3)
ASRS total ^e^	25.8 (14.1)	18.2 (9.0)	41.5 (11.4)	24.3 (12.5)	39.2 (10.6)
ASRS impulsivity ^f^	11.4 (6.3)	10.1 (5.1)	NA	10.4 (6.4)	19.0 (5.3)
ASRS inattention ^f^	11.6 (7.4)	7.9 (4.9)	NA	13.9 (7.1)	20.2 (7.2)
SSP impulsivity ^g^	16.3 (3.2)	15.9 (2.8)	NA	16.9 (3.6)	NA
SSP social desirability ^g^	20.1 (2.6)	20.6 (2.7)	NA	19.4 (2.4)	NA

OCDS – Obsessive Compulse Drinking Scale; BIS – Barratt Impulsiveness Scale; MADRS-S - Montgomery-Åsberg Depression Self Rating Scale; ASRS - Adult ADHD Self-report Scale; SSP - Swedish universities Scale of Personality. NA – no data available

^a^ School year data available from 139 participants^b^ OCDS data available from 149 participants

^c^ Drinking data available from 178 participants

^d^ MADRS-S data available from 188 participants

^e^ ASRS total score data available from 201 participants

^f^ ASRS subscales data available from 135 individuals

^g^ SSP data available from123 individuals

### Internal consistency reliability

For all the 30 swe-BIS items in the full study sample, the Cronbach’s alpha was .89 [95% CI .87–.91], with slightly lower coefficients for each of the subscales of attentional (.79 [.76–.83]), motor (.72 [.67–.77]) and non-planning impulsiveness (.77 [.72–.81]). Similar but reduced Cronbach’s alpha coefficients were found for the HC and the different patient populations (Table 2A).

**Table 2:** Internal consistency assessed by Cronbach’s alpha [95% confidence intervals] (A) and test-retest Pearson correlations (B) for the Swedish version of the Barratt Impulsiveness Scale (swe-BIS) administered to healthy controls, patients with amphetamine use disorder (AMPH), alcohol use disorder (AUD) and attention deficit hyperactive disorder (ADHD)

	A. Internal consistency (Cronbach’s alpha)				
	Full sample (*n* = 273)	Healthy controls (*n* = 113)	AMPH (*n* = 37)	AUD (*n* = 97)	ADHD (*n* = 26)
Swe-BIS all items	.89 [.87–.91]	.78 [.72–.89]	.87 [.82–.93]	.82 [.77–.87]	.87 [.80–.94]
Swe-BIS attention	.79 [.76–.83]	.60 [.49–.71]	.70 [.56–.85]	.72 [.64–.80]	.63 [.42–.85]
Swe-BIS motor	.72 [.67–.77]	.50 [.36–.64]	.68 [.53–.83]	.62 [.51–.73]	.71 [.56–.87]
Swe-BIS non-planning	.77 [.72–.81]	.70 [.62–.79]	.71 [.58–.85]	.64 [.54–.75]	.81 [.70–.92]
	B. Test-retest Pearson correlations across subscales				
	Swe-BIS total score (re-rest)	Swe-BIS attention (re-test)	Swe-BIS motor (re-test)	Swe-BIS non-planning (re-test)	
Swe-BIS total score (test)	.78 *P* < 0.001				
Swe-BIS attention (test)		.63 *P* < .001	.18 *P* = .16	.36 *P* = .005	
Swe-BIS motor (test)		.39 *P* = .002	.54 *P* < .001	.26 *P* = .04	
Swe-BIS non-planning (test)		.48 *P* < .001	.38 *P* = .003	.77 *P* < .001	

### Test-retest reliability

Among the HC, a total of 62 individuals filled in the swe-BIS questionnaires on two occasions, approximately 1 week between completion of the questionnaires. There was a statistically significant test-retest correlation for swe-BIS total score (*r* = .78; *p* < .001), as well as for the subscales of attentional (*r* = .63; *p* < .001), motor (*r* = .54; *p* < .001) and non-planning (*r* = .77; *p* < .001) impulsiveness. The test-retest correlations were greater within each subscale, compared to the correlations across different subscales (Table 2B).

**Fig. 1:**
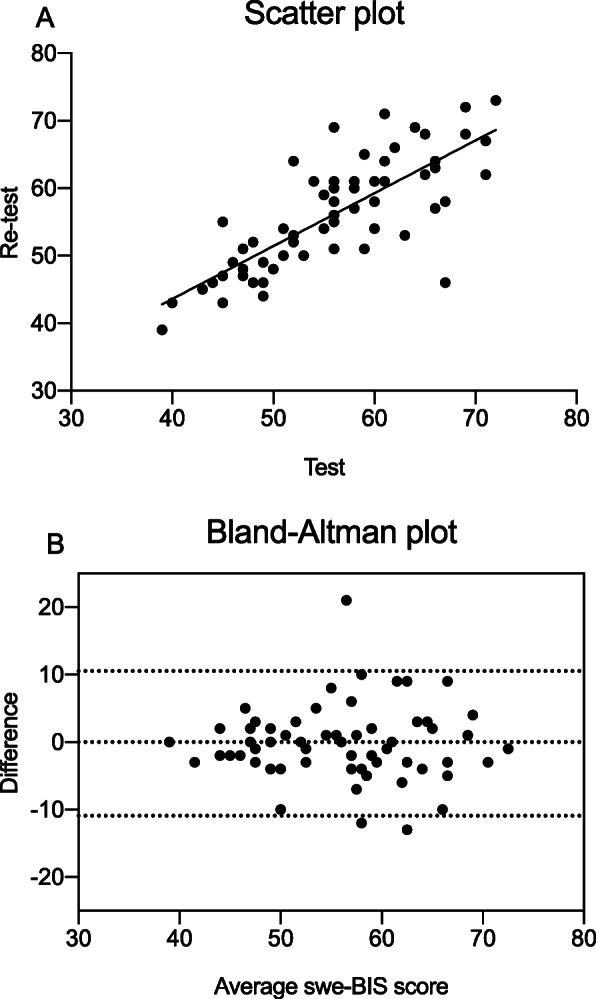
The correlation between test and retest result on the Swedish version of the Barratt Impulsiveness Scale (swe-BIS) was 0.78 (**A**). The Bland-Altman plot presents the mean total swe-BIS score across both tests against the mean difference between test sessions (**B**). Three individuals (4.8%) had mean difference scores outside the limits of + − 2 standard deviations of the mean difference, indicating good agreement between test and re-test

The test-retest results are presented as correlation between first and second test score (Fig. 1A) and a Bland-Altman graph plotting the mean swe-BIS score against difference between test sessions (Fig. 1B). These plots illustrate that there seems to be slightly greater differences between test sessions with higher test scores. Furthermore, in the Bland-Altman plot the mean average difference was close to zero (− 0.2), and only three individuals out of 62 (4.8%) had data points that fell outside the 95% CI of the mean difference.

### Validity based on response processes

The HC had lower swe-BIS scores compared to the patient sample as a whole, for the BIS total score (HC: 56.2(8.9); Patients: 68.3(14.3); t(267.3) = − 8.6; *p* < .001) as well as the attentional (HC: 12.9(2.8); Patients: 16.9(4.6); t(265.2) = − 9.1; *p* < .001), motor (HC: 20.7(4.1); Patients: 24.3(5.3); t(269.5) = − 6.4; *p* < .001) and non-planning (HC: 22.7(4.5); Patients: 27.1(6.4); t(271.0) = − 6.6; *p* < .001) subscales.

**Fig. 2:**
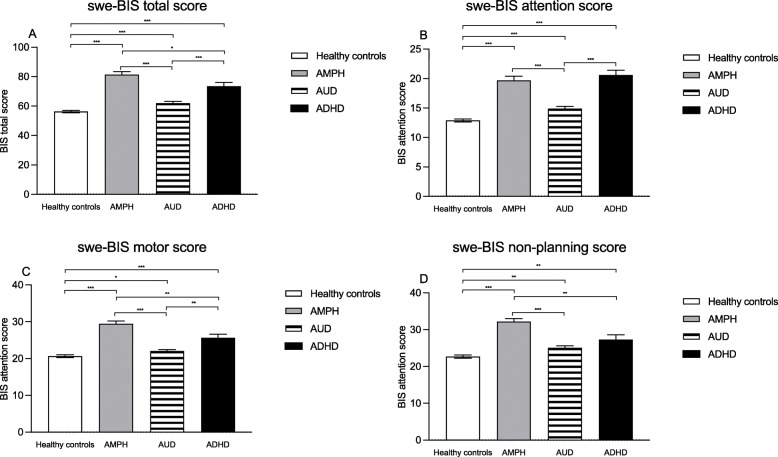
Comparisons between healthy controls and patients with amphetamine use disorder (AMPH), alcohol use disorder (AUD) and attention deficit hyperactivity disorder (ADHD) on the Swedish version of the Baratt Impulsiveness Scale (swe-BIS) total score (**A**) and subscales of attention (**B**), motor (**C**) and non-planning (**D**) impulsiveness. * *p* < .05; ** *p* < .01; *** *p* < .001 for the post hoc comparisons.

The ANOVA analysis including all subgroups (HC, AUD, AMPH, ADHD) of the swe-BIS total score indicated a statistically significant effect of group (F(3,76.4) = 49.6; *p* < .001; Fig. 2A). Post hoc comparisons found statistically significant differences for all comparisons between all four groups: The HC had significantly lower swe-BIS total score (HC mean: 56.2(8.9)) than patients with AMPH (Mean: 81.3; *p* < .001), AUD (Mean: 62.0; *p* < .001) and ADHD (Mean: 73.5; *p* < .001). The AMPH group had highest score among the patients scoring significantly higher than both AUD (*p* < .001) and ADHD (*p* = .024), who in turn had higher scores than the AUD group (*p* < .001). A similar pattern of results was found for all the swe-BIS subscales (Fig. 2B-D).

### Evidence of convergent and divergent validity

The swe-BIS total score exhibited statistically significant correlations with the other impulsivity questionnaires i.e., ASRS (*r* = .81; *p* < .001), ASRS impulsivity (*r* = .67; *p* < .001), ASRS inattention (*r* = .70; *p* < .001) and SSP impulsivity subscale (*r* = .62; *p* < .001). Notably, the correlations were lower between swe-BIS total score and the non-impulsivity related scales i.e., MADRS-S (*r* = .46; *p* < .001), OCDS (*r* = .35; *p* < .001) and SSP social desirability (*r* = −.20; *p* = .024).

### Confirmatory factor analysis

The Mardia’s Multivariate Normality Test was highly statistically significant for both skewness and kurtosis, signaling that data was not multivariate normal (z-kurtosis = 20.62, *p* < .001). We therefore used the MLR (robust Maximum Likelihood) estimation method in all CFA of the swe-BIS scale. The results from the CFA of five different models of the swe-BIS scale are summarized in a number of common model fit indices in Table 3.

**Table 3:** Confirmatory Factor Analysis (CFA) results with model fit indices of five models of the Swedish version of the Barratt Impulsiveness Scale (swe-BIS)

Model	χ^2^	Df	RMSEA [90% CI]	SRMR	CFI	TLI	AIC
1-factor model	1166.436	405	0.083 [0.078–0.088]	0.084	0.691	0.668	19,102.624
2-factor model, 1st version^a^	1137.630	404	0.082 [0.076–0.087]	0.083	0.702	0.679	19,071.382
2-factor model, 2nd version^b^	1130.500	404	0.081 [0.076–0.086]	0.084	0.705	0.682	19,061.207
2-factor model, 3rd version^c^	1152.555	404	0.082 [0.077–0.088]	0.084	0.696	0.673	19,087.222
3-factor model^d^	1111.630	402	0.080 [0.075–0.086]	0.084	0.712	0.688	19,042.103

*χ*^*2*^ chi square value, *RMSEA* Root mean squared error of approximation, *SRMR* Standardized root mean squared residual, *CFI* Comparative fit index, *TLI* Tucker-Lewis index, *AIC* Akaike’s information criterion

^a^Attentional – Motor/Nonplanning

^b^Motor – Attentional/Nonplanning

^c^Nonplanning – Attentional/Motor

^d^The model with best fit

According to RMSEA, all the models have a mediocre fit (0.08–0.10) and none of them have an upper limit of the confidence interval below 0.08 which indicates a reasonable fit. No model showed a satisfactory value on CFI and TLI (> 0.90) or acceptable SRMR value (< 0.08), even though the 3-factor model had the best values among tested models. The AIC value was also lowest for the 3-factor model. The best model for our data, based on an assessment of all the fit indices presented in Table 3 was thus the 3-factor model. Twenty-one of the thirty items had good factor loadings (≥ 0.40) in the 3-factor model. Of the nine items with a lower factor loading (< 0.40), only item 24 (0.313) was from the attentional subscale, while item 15 (0.311), 27(0.353) and 29 (0.355) were from the non-planning subscale. Finally, in the motor subscale item 3 (0.275), 16 (0.253), 21 (0.268), 23 (0.301) and 30 (0.335) had lower factor loading. All individual item factor loadings for the 3-factor model are presented in the supplementary material Table S2.

## Discussion

In the current study, the swe-BIS was found to have overall satisfying psychometric properties when administered to both healthy individuals and clinical populations with AUD, AMPH and ADHD. Importantly, indices of both reliability and validity were in general good to excellent, suggesting that the scale can be administered to both healthy individuals as well as clinical patient populations with externalizing disorders.

The swe-BIS exhibited excellent internal consistency, as assessed by a Cronbach’s alpha coefficient of .89 in the full sample, and .78–.87 in the different patient populations. There are different reports on cut-off values for the Cronbach’s alpha, but often values in the range of .70–.95 are considered acceptable [47]. For the different subscales, the values of Cronbach’s alpha were in general also acceptable with values of .72–.79 in the full sample. These results are similar to previously reported Cronbach’s alpha values for several adaptations to different languages, as discussed in the review by Stanford and colleagues [7].

The correlation between test sessions was .78, and the test-retest correlations were greater within each subscale compared to across the subscales, indicating acceptable test-retest reliability for both the total score and subscales. Finally, the Bland-Altman plot illustrated that only a minority of participants had mean-difference scores outside the limits of + − 2 standard deviations of the mean difference, indicating good agreement between test sessions. Importantly, since one of the completed BIS questionnaires was completed at home, these results also support that it is feasible to administer the scale outside of the clinic without supervision of research staff. Taken together, the Cronbach’s alpha and test-retest results indicate that the swe-BIS has good reliability, which is in line with conclusions from previous studies of the BIS-11 [10].

The validity analyses found that the swe-BIS was able to discriminate not only between HC and patients in general, but also was able to differentiate between different clinical patient populations. The findings of elevated self-rated impulsiveness in SUD and ADHD are in line with several previous studies [15–19]. Notably, the BIS scores for the AUD group in the current study was significantly lower than the AMPH patients. This is likely in part explained by the fact that the different patient populations had different severity of dependence. While the AUD patients in general had a stable social situation and a wider distribution of number of DSM-IV alcohol dependence criteria fulfilled (range 3–7; Mean: 5.0 (1.2)), the AMPH group had more social problems and consistently severe levels of substance dependence (range 5–7; Mean: 6.7 (0.7)). These results are in line with previous studies that have found higher BIS scores in early onset AUD thought to reflect a more severe form of the disorder [48]. Finally, we found that the swe-BIS score correlated to a higher degree with other indices of impulsivity, and to a lesser degree with clinical self-rating scales measuring depressive symptoms, craving or other personality traits. Taken together the swe-BIS exhibited satisfying validity and our results suggest that it can identify group differences regarding impulsiveness within and between different externalizing disorders such as SUD and ADHD. Furthermore, our results from the convergent/divergent validity analyses suggest that the swe-BIS captures specific aspects of impulsiveness, and not a general propensity of responding high scores on questionnaires in general.

The confirmatory factor analysis indicated that none of the models achieved a good fit to the observed data, based on different fit indices such as CFI, TLI or SRMR. Of all the tested models however, the 3-factor model had the lowest AIC value, suggesting that even though far from perfect the 3-factor model indeed provided the best fit to the observed data. However, it is important to note that 9 items had a poor factor loading (< 0.4) and these items related to all three factors, but mainly to motor (five items) and non-planning (three items) subscales. In future revisions of the swe-BIS these items may be considered to revise or retranslate in order to improve the factor structure of the scale. It is important however, that almost all studies of different BIS versions have found different results for the optimal factor structure of the BIS scale [10], likely reflecting cultural differences in interpretation of certain items and the overall heterogeneity of the impulsiveness construct.

While the current study has several strengths, such as inclusion of different patient populations and detailed assessment (and exclusion) of severe psychiatric co-morbidity, there are some important limitations in need of discussion. First of all, sample size was limited especially for the patient groups of AMPH and ADHD, so the estimates in these samples are less certain than for the HC and AUD groups. Second, test-retest data was only available for a subsample of the HC (*n* = 62). The results regarding test-retest are therefore not necessarily generalizable to the clinical patient populations. Furthermore, the timing of the test-retest was on average 1 week, so no conclusions regarding long-term test-retest and agreement can be made based on this study. Finally, data for the convergent and divergent validity analyses were only available in a subsample of HC and AD patients (*n* = 123), so therefore we cannot infer that the same results hold for AMPH or ADHD.

## Conclusions

In summary, the swe-BIS exhibited good to excellent psychometric properties regarding reliability and validity and can be utilized in healthy individuals and patients with externalizing disorders such as SUD and ADHD. While far from optimal, the 3-factor solution provided the best model fit suggesting that the three subscales of motor, non-planning and attentional impulsiveness can be analyzed albeit with caution. In order to investigate the general validity of the swe-BIS further, future studies could include patients with other psychiatric disorders (e.g., affective disorders or personality disorders) or neurological disorders (e.g., Parkinson’s disease) where the disorder or treatment response might be characterized by impulsive behavior.

## Supplementary Information

**Additional file 1: Table S1.** The translated version of the Swedish version of the Barratt Impulsiveness Scale (swe-BIS). **Table S2.** Individual item factor loadings for the best fitting 3-factor model for the Swedish version of the Barratt Impulsiveness Scale (swe-BIS).

## Data Availability

The datasets generated and/or analysed during the current study are not publicly available due to individual privacy but are available in summary/group level form from the corresponding author on reasonable request.

## Abbreviations

ADHD: Attention Deficit Hyperactivity Disorder
AIC: Akaike’s Information Criterion
AMPH: Amphetamine Use Disorder
ASRS: The World Health Organization Adult ADHD Self-report Scale
AUD: Alcohol Use Disorder
BIS: Barratt Impulsiveness Scale
CFA: Confirmatory Factor Analyses
CFI: Comparative Fit Index
HC: Healthy Controls
MADRS-S: Montgomery-Åsberg Depression Self-Rating Scale
MLR: Robust Maximum Likelihood
OCDS: The Obsessive Compulsive Drinking Scale
RMSEA: Root Mean Squared Error of Approximation
SRMR: Standardized Root Mean Squared Residual
SSP: The Swedish Universities Scales of Personality
SUD: Substance Use Disorder
Swe-BIS: Swedish version of the Barratt Impulsiveness Scale
TLFB: Time Line Follow Back
TLI: Tucker-Lewis Index
