# Supplementary material for: Self-rated impulsivity in healthy individuals, substance use disorder and ADHD: psychometric properties of the Swedish Barratt impulsiveness scale
Source: BMC Psychiatry. 2021 Sep 18;21:458. doi: 10.1186/s12888-021-03462-1 (PMC8449879; doi:10.1186/s12888-021-03462-1)
Supplement: Supplementary file 1 — Additional file 1: Table S1. The translated version of the Swedish version of the Barratt Impulsiveness Scale (swe-BIS). Table S2. Individual item factor loadings for the best fitting 3-factor model for the Swedish version of the Barratt Impulsiveness Scale (swe-BIS). [file 12888_2021_3462_MOESM1_ESM.docx]

**Supplementary material**

**Table S1.** The translated version of the Swedish version of the Barratt Impulsiveness Scale (swe-BIS).

| 1 Jag planerar aktiviteter noggrant. |
| --- |
| 2 Jag gör saker utan att tänka. |
| 3 Jag fattar beslut snabbt. |
| 4 Jag tar dagen som den kommer. |
| 5 Jag är ouppmärksam. |
| 6 Jag har “rusande” tankar. |
| 7 Jag planerar resor i god tid. |
| 8 Jag har god självkontroll. |
| 9 Jag har lätt för att koncentrera mig. |
| 10 Jag sparar regelbundet. |
| 11 Jag “skruvar på mig under pjäser eller föreläsningar. |
| 12 Jag tänker efter noga. |
| 13 Jag planerar för anställningstrygghet. |
| 14 Jag säger saker utan att tänka. |
| 15 Jag gillar att fundera på komplicerade problem. |
| 16 Jag byter jobb. |
| 17 Jag agerar “impulsivt”. |
| 18 Jag blir lätt uttråkad när jag löser tankeproblem. |
| 19 Jag agerar på stundens ingivelse. |
| 20 Jag är eftertänksam. |
| 21 Jag byter bostad. |
| 22 Jag gör impulsköp. |
| 23 Jag kan bara tänka på en sak i taget. |
| 24 Jag byter hobby. |
| 25 Jag spenderar mer än jag tjänar. |
| 26 Jag har ofta ovidkommande tankar. |
| 27 Jag är mer intresserad av nuet än av framtiden. |
| 28 Jag känner mig rastlös på bio eller föreläsningar. |
| 29 Jag gillar tankenötter. |
| 30 Jag är framtidsinriktad. |

**Table S2**. Individual item factor loadings for the best fitting 3-factor model for the Swedish version of the Barratt Impulsiveness Scale (swe-BIS).

|  | Factor loading |
| --- | --- |
| **Attentional impulsiveness** |  |
| Swe-BIS item 5 | 0.510 |
| Swe-BIS item 6 | 0.636 |
| Swe-BIS item 9 | 0.778 |
| Swe-BIS item 11 | 0.551 |
| Swe-BIS item 20 | 0.487 |
| Swe-BIS item 24 | 0.313 |
| Swe-BIS item 26 | 0.660 |
| Swe-BIS item 28 | 0.632 |
| **Motor impulsiveness** |  |
| Swe-BIS item 2 | 0.670 |
| Swe-BIS item 3 | 0.275 |
| Swe-BIS item 4 | 0.460 |
| Swe-BIS item 16 | 0.253 |
| Swe-BIS item 17 | 0.757 |
| Swe-BIS item 19 | 0.786 |
| Swe-BIS item 21 | 0.268 |
| Swe-BIS item 22 | 0.644 |
| Swe-BIS item 23 | 0.301 |
| Swe-BIS item 25 | 0.587 |
| Swe-BIS item 30 | 0.335 |
| **Non-planning impulsiveness** |  |
| Swe-BIS item 1 | 0.510 |
| Swe-BIS item 7 | 0.563 |
| Swe-BIS item 8 | 0.620 |
| Swe-BIS item 10 | 0.537 |
| Swe-BIS item 12 | 0.652 |
| Swe-BIS item 13 | 0.517 |
| Swe-BIS item 14 | 0.578 |
| Swe-BIS item 15 | 0.311 |
| Swe-BIS item 18 | 0.633 |
| Swe-BIS item 27 | 0.353 |
| Swe-BIS item 29 | 0.355 |
